# A Novel *AXIN2* Missense Mutation Is Associated with Non-Syndromic Oligodontia

**DOI:** 10.1371/journal.pone.0138221

**Published:** 2015-09-25

**Authors:** Haochen Liu, Tingting Ding, Yuan Zhan, Hailan Feng

**Affiliations:** 1 Department of Prosthodontics, Peking University School and Hospital of Stomatology, Beijing, China; 2 The Third Dental Center, Peking University School and Hospital of Stomatology, Beijing, China; New York Medical College, UNITED STATES

## Abstract

Oligodontia is defined as the congenital absence of six or more permanent teeth, excluding the third molars. Oligodontia may contribute to masticatory dysfunction, speech alteration, aesthetic problems and malocclusion. Numerous gene mutations have been association with oligodontia. In the present study, we identified a *de novo AXIN2* missense mutation (c.314T>G) in a Chinese individual with non-syndromic oligodontia. This mutation results in the substitution of Val at residue 105 for Gly (p.Val105Gly); residue 105 is located in the highly conserved regulator of G protein signaling (RGS) domain of the AXIN2 protein. This is the first report indicating that a mutation in the RGS domain of AXIN2 is responsible for non-syndromic oligodontia. Our study supports the relationship between *AXIN2* mutation and non-syndromic oligodontia and extends the mutation spectrum of the *AXIN2* gene.

## Introduction

Tooth agenesis, the congenital lack of one or more permanent and/or deciduous teeth, is a well-recognized morphologic anomaly in humans. The absence of one to six teeth (excluding the third molars), more than six teeth (excluding the third molars), and the complete absence of teeth have been termed hypodontia, oligodontia, and anodontia, respectively [1]. Tooth agenesis can occur either as isolated findings or as part of a syndrome [2]. Both environmental and genetic factors can cause tooth agenesis, with genetic factors representing by far the most common cause of tooth agenesis [3].

Numerous genes have been implicated in tooth development and in theory, any of these genes may cause tooth agenesis. To this day, mutations in nine genes (*MSX1*, *PAX9*, *AXIN2*, *WNT10A*, *EDA*, *EDAR*, *EDARADD*, *NEMO* and *KRT17*) have been associated with non-syndromic oligodontia [4–13]. The transcription factor genes *MSX1* and *PAX9* were the first and second genes to be identified in non-syndromic oligodontia [7,14]. The MSX1 and PAX9 proteins are responsible for the interaction between dental tissues and are essential for the establishment of the odontogenic potential of the mesenchyme [15]. AXIN2 is an intracellular antagonist of Wnt signaling and WNT10A is a member of the Wnt family. Both *AXIN2* and *WNT10A* can cause non-syndromic oligodontia [16–17]. *EDA* is a well-known gene that causes ectodermal dysplasia; however, several studies also reported that mutations in *EDA* can cause non-syndromic oligodontia[4,9,18–19]. *EDARADD* is a downstream signaling mediator of *EDA* and one study reported that a mutation in this gene led to non-syndromic oligodontia[6].

In the present study, we investigated six genes (*MSX1*, *PAX9*, *AXIN2*, *WNT10A*, *EDA* and *EDARADD*) in a patient with sporadic non-syndromic oligodontia. Finally, we identified a *de novo AXIN2* missense mutation, which was located in a highly conserved region of the encoded protein.

## Materials and Methods

### Study Individuals and Samples

Subjects of this study were of Chinese descent. The 8-year-old male was recruited from among the patients referred to the Peking University School of Stomatology (the 3rd Dental Center for diagnostic evaluation and treatment of oligodontia). A pedigree was constructed by clinical examination of available family members and through interviews. An experienced dentist determined the status of dentition through oral and panoramic radiographic examinations for the proband and his parents. Peripheral blood samples were taken from the proband and his parents, in addition to 100 unrelated healthy individuals who were not affected with tooth agenesis or other craniofacial abnormalities (control group). Written informed consent was obtained from all the participants and the parents on the behalf of the minors or children participants. The study protocol and subject consent were approved by the Institutional Review Board of Peking University School and Hospital of Stomatology (No.IRBSS2014NNSF01).

### Mutation Analysis for Candidate Genes

Genomic DNA was isolated from peripheral blood samples by use of the Biotek DNA Mini-kit (Biotek, Beijing, China), following the manufacturer’s instructions. The *MSX1* (NM_002448), *PAX9* (NM_006194), *AXIN2* (NM_004655), *WNT10A* (NM_025216), *EDA* (NM_001399) and *EDARADD* (NM_080738) genes were selected for genetic analysis, and their exons and exon-intron junctions were amplified by polymerase chain reaction (PCR). Direct DNA sequencing was performed using ABigDye terminator v3.1 (Applied Biosystems, Foster City, USA) and a 3730 DNA sequencer (Applied Biosystems). PCR primers and conditions were consistent with previous studies [6,9,17,20–23].

### Conservation Analysis and WebLogo Analysis

Multiple-species amino acid sequence alignment and WebLogo analysis of the AXIN2 protein (NP_004646.3) were carried out separately by ClustalX 2.1 and WebLogo Version 2.8.2 (http://weblogo.berkeley.edu) [24]. AXIN2 sequences from zebrafish to human were obtained from ENSEMBL.

### Structural Analysis

Three-dimensional models of the wild-type regulator of G protein signaling (RGS) domain of the AXIN2 protein and the mutant RGS (p.Val105Gly) domain were designed using the Phyre threading software (www.sbg.bio.ic.ac.uk/phyre/) [25], based on primary sequence conservation and known protein structures. The models were manipulated using the PyMOL software (PyMOL Molecular Graphics System, DeLano Scientific, San Carlos, CA).

## Results

### Clinical findings

**Fig 1:**
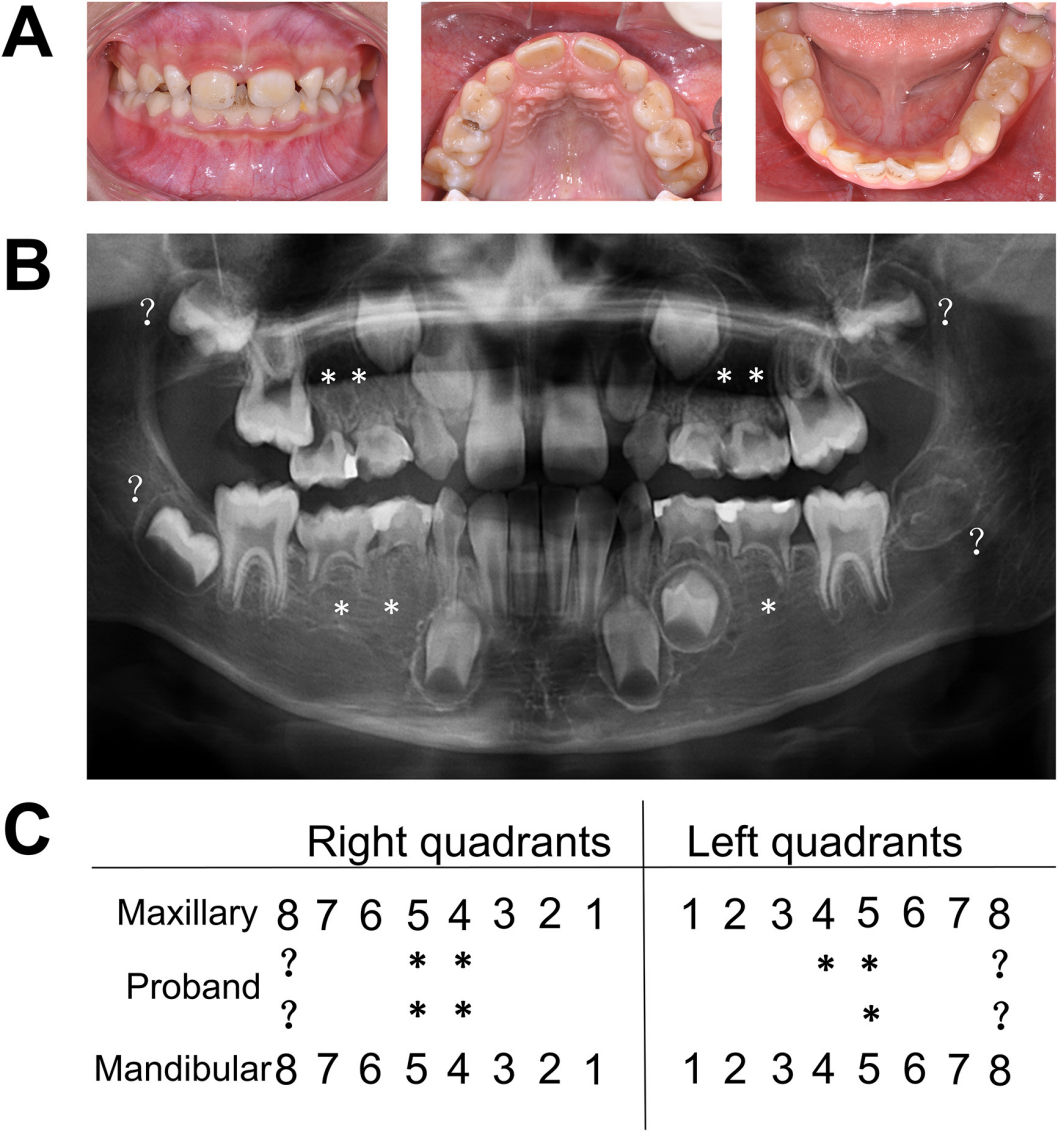
Clinical characteristics of the proband with non-syndromic oligodontia. (A) Clinical phenotype of the proband. (B) Panoramic radiograph of the participant. (C) Schematic presentation of congenitally missing teeth of the proband. * Position of a missing tooth;? Undetermined position of a missing tooth.

The proband was an 8-year-old male with a normal appearance. Clinical and radiographic examinations revealed that, in addition to retained deciduous teeth, the patient was missing a total of seven permanent teeth excluding third molars, all of them premolars (Fig 1). The incisors, canines, first molars and second molars were normal. No tooth germ for third molars was detected. Considering his young age, a diagnosis could not yet be made in relation to the lack of third molars. The proband’s facial features, skin, hair and nails appeared normal. Also, he reported normal sweating and lachrymal secretion and denied intolerance to heat, or susceptibility to respiratory tract infections. His parents’ dentition was normal, and they reported no family history of tooth agenesis, ectodermal abnormalities or cancer.

### Mutation analysis

**Fig 2:**
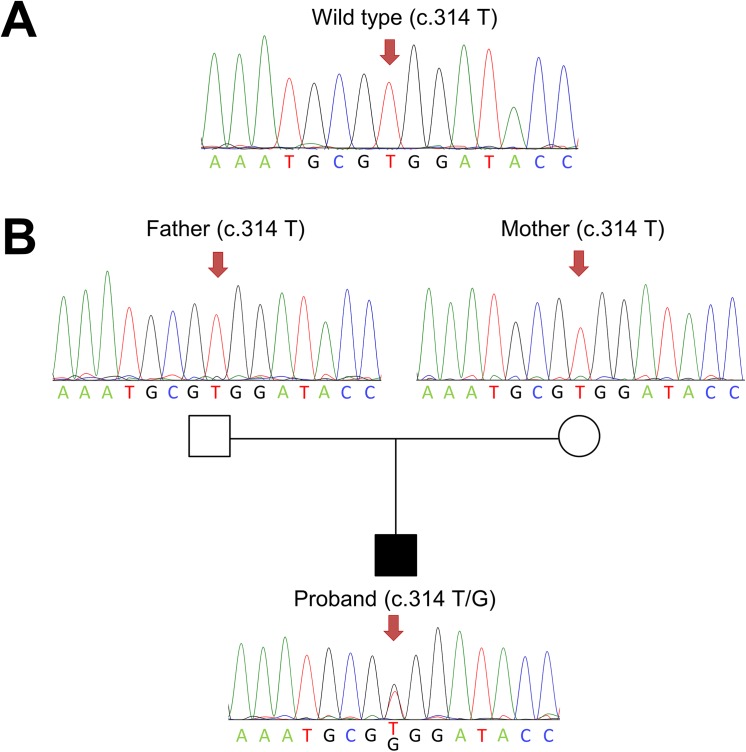
Sequence analyses of the *AXIN2* gene. (A) A normal control shows the wild-type genotype. (B) A *de novo* heterozygous mutation, c.314T>G, was found in the proband, but not in his parents.

Following mutation analysis of six genes (*MSX1*, *PAX9*, *AXIN2*, *WNT10A*, *EDA* and *EDARADD*), we found a novel missense mutation in *AXIN2* in the proband. The nucleotide sequence showed a heterozygous T to G transition at nucleotide 314 (c.314T>G) of the coding sequence in exon 2 of *AXIN2* (Fig 2), which resulted in the substitution of Val at residue 105 for Gly. We have submitted the information of this *AXIN2* mutation to ClinVar database in NCBI (www.ncbi.nlm.nih.gov/clinvar/; Accession number: SCV000223708). The proband’s parents did not carry the mutation (Fig 2). Also, the mutation was not detected in 100 normal controls (Fig 2). Therefore, this mutation is likely to have occurred *de novo*.

### Conservation analysis

**Fig 3:**
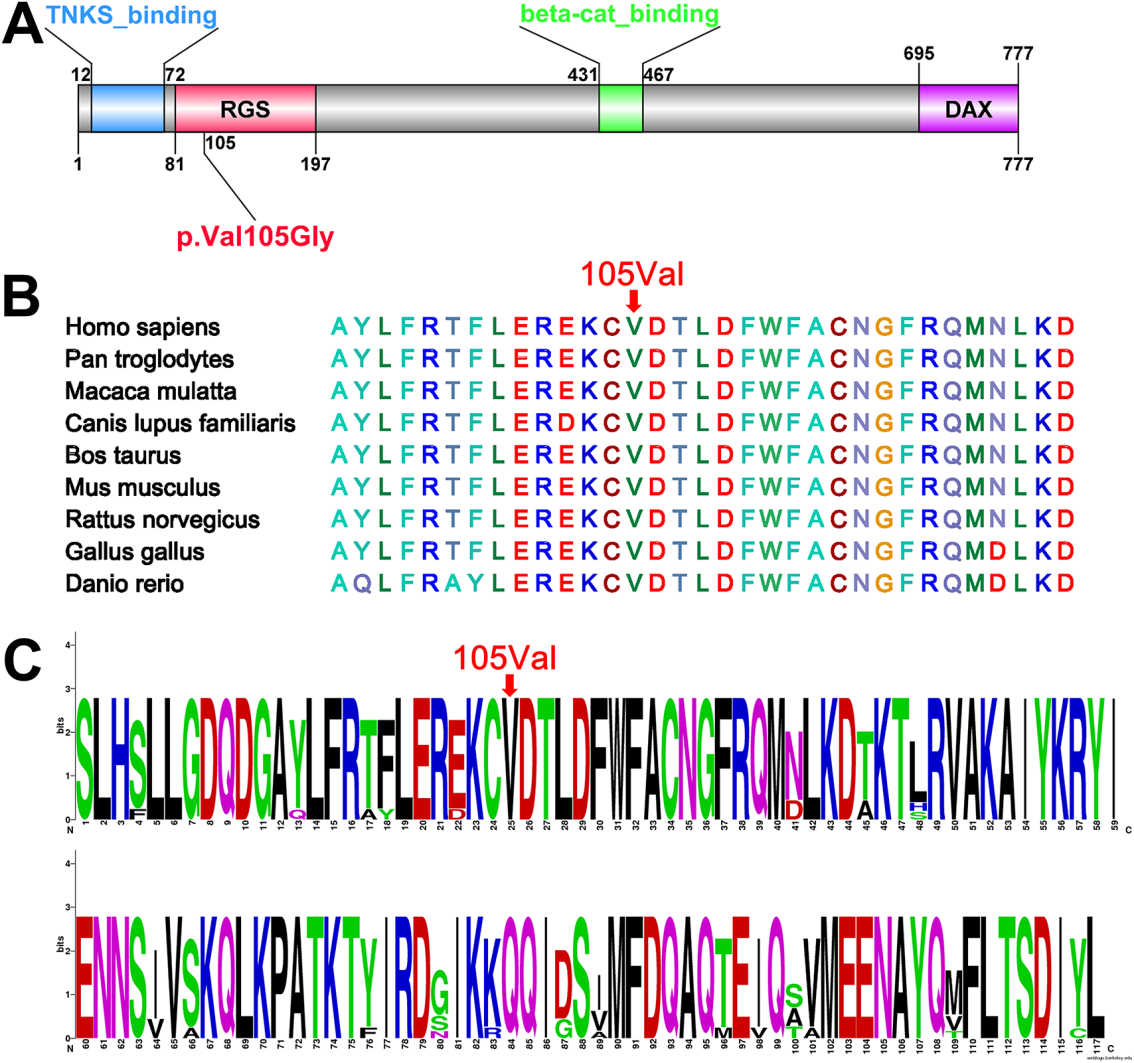
Structure and conservation analysis of the p.Val105Gly mutation in AXIN2. (A) The AXIN2 protein is 777 AA long with 4 conserved domains. The 105th AA position is mutated in the proband Val105Gly which resides in the RGS domain. (B) Evolutionary conservation analysis revealed that the Val105 site is conserved from zebrafish to humans. There are 28 out of 33 AA near the Val105 site that are conserved, this is a very high degree of conservation. (C) WebLogo analysis showed that the RGS domain (from zebrafish to humans) was relatively conserved. TNKS_binding: Tankyrase binding N-terminal segment of axin; RGS: Regulator of G protein signaling (RGS) domain; DAX: Domain present in Dishevelled and Axin.

Val105 is located at the RGS domain of the AXIN2 protein (Fig 3). Evolutionary conservation analysis revealed that the Val105 site is conserved from zebrafish to humans (Fig 3), suggesting that Val105 may play an important role in the function of the RGS domain. To evaluate the conservation of the RGS domain of the AXIN2 protein, we carried out WebLogo analysis (http://weblogo.berkeley.edu) and compared the conservation of all amino acids of the RGS domain from zebrafish to humans based on multiple alignments. We found that the RGS domain was relatively conserved (Fig 3).

### Structural Analysis

**Fig 4:**
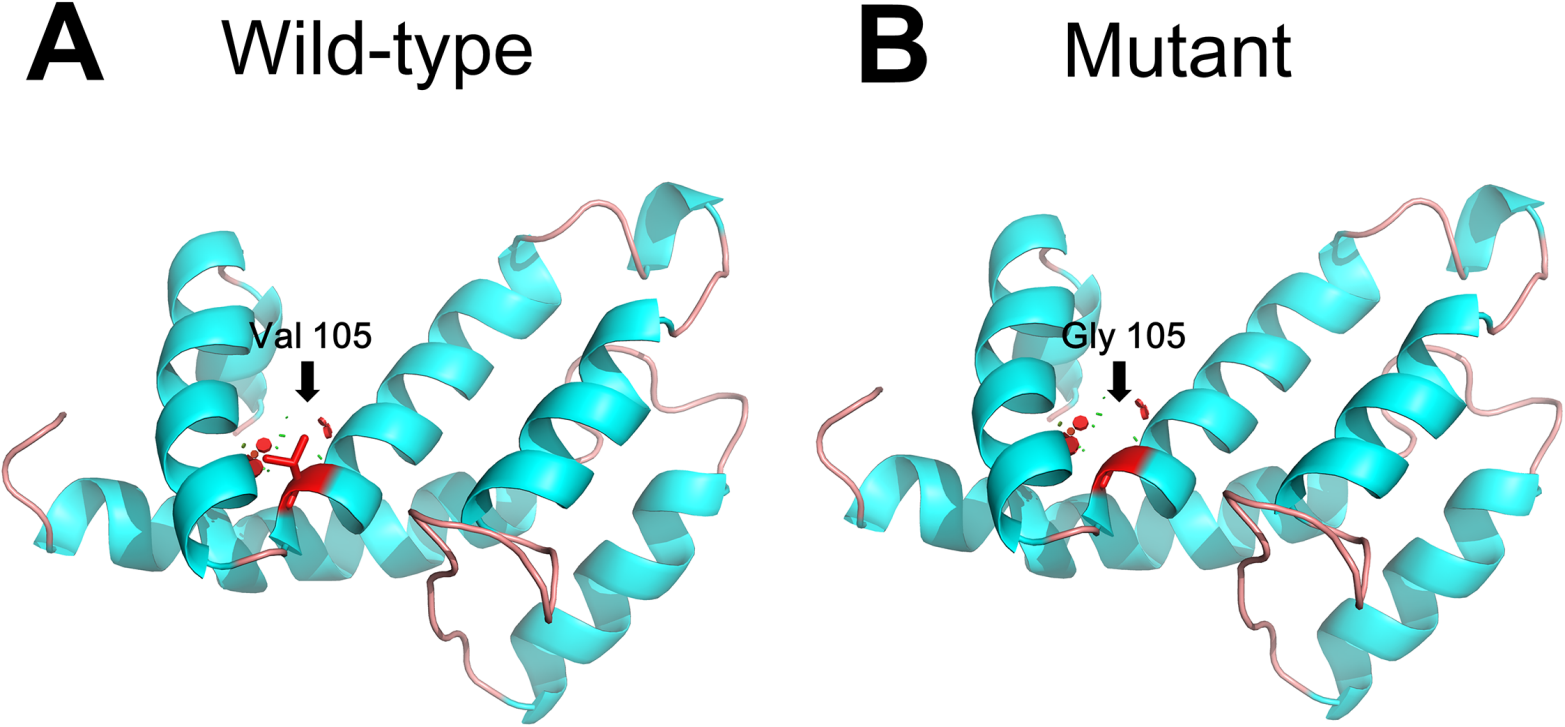
Structural analysis of the RGS domain of the AXIN2 protein. (A) Location of the Val105 residues within the RGS domain of AXIN2. (B) Location of the Gly105 residues within the RGS domain of AXIN2.

Structural analysis predicted that the 3D structures of the wild-type and mutant RGS domain of AXIN2 are different at residue 105 (Fig 4), which suggests that the p.Val105Gly substitution may affect the structure of the RGS domain of the AXIN2 protein.

## Discussion

Our findings indicate that the novel heterozygous transition found in *AXIN2* (c.314T>G) might be responsible for the presentation of non-syndromic oligodontia in the proband. This mutation, which results in a Val105Gly substitution, is localized to the regulator of G protein signaling (RGS) domain found in the Axin protein. RGS domains are protein structural domains that activate GTPases via heterotrimeric G-protein alpha-subunits; RGS proteins have been conserved in evolution [26]. This missense mutation (c.314T>G) may affect the function of the RGS domain of the AXIN2 protein, leading to oligodontia.

In families with severe tooth agenesis and in several mutant mouse lines, the phenotypes are variable in expression, presumably due to the effects of modifying genes and other genetic backgrounds as well as postgenetic factors [27]. In tooth agenesis, this variation in effect of major genetic factors can be reflected as incomplete penetrance. In this study, the proband’s parents displayed normal dentition and reported no family history of tooth agenesis or other ectodermal abnormalities. This may suggest incomplete penetrance of oligodontia in this family. However, the novel mutation was identified only in the proband, and his parents did not carry the mutation (Fig 2). Moreover, the highly conserved character of the mutated nucleotide (Fig 3), as well as the number of analyzed control alleles (100), precludes the possibility that this transition is a rare polymorphism of *AXIN2*. Hence, we confirm that this missense mutation is a *de novo* germline mutation. A number of studys have reported SNPs in *AXIN2* associated with tooth agenesis [23,28–29], Mu *et al*. [23] presented 7 SNPs in the *AXIN2*, and 3 SNPs were in exons. We did not find any SNPs in *AXIN2* in the proband and his parents. It’s seemed that SNPs in *AXIN2* is not a risk factor for this family.

Five conserved signaling pathways, including the fibroblast growth factors (Fgf), bone morphogenetic proteins (Bmp), ectodysplasin (Eda), wingless-related (Wnt) and sonic hedgehog (Shh) pathways, play a critical role throughout tooth development [30]. Inactivation of any of these pathways results in early tooth developmental arrest in mice. Mutations in these pathways have also been identified in human patients with tooth agenesis. The MSX1 and PAX9 transcription factors are involved in the Bmp and Fgf pathways during tooth development [31]. The Eda pathway is active during the development of ectodermal organs, including teeth, hairs, feathers, and mammary glands and was discovered by studying human patients affected by anhidrotic/hypohidrotic ectodermal dysplasia. It comprises three main gene products: EDA, a ligand that belongs to the tumor necrosis factor (TNF)-α family, EDAR, a receptor related to the TNFα receptors, and EDARADD, a specific adaptor [32]. Recently, *WNT10A*, belonging to the Wnt pathway, became a focal candidate gene for tooth agenesis. *WNT10A* was identified as a causal gene of autosomal recessive ectodermal dysplasia or isolated tooth agenesis [33–35]. Moreover, Pakeeza *et al*.[36] stated that *WNT10A* mutations account for one-quarter of population-based isolated oligodontia, and Van den Boogaard *et al*.[8] reported that mutations in *WNT10A* are present in more than half of isolated hypodontia cases. In the present study, we did not identify any mutation in *WNT10A*, but one was identified in *AXIN2*. AXIN2 is an intracellular antagonist of the Wnt signaling pathway. Thus, this missense mutation (c.314T>G) in *AXIN2* may affect tooth development via the Wnt pathway.

To date, four studies reported seven *AXIN2* mutations associated with syndromic or non-syndromic tooth agenesis [6,16,21,37]. Among the seven, two mutations caused syndromic tooth agenesis: in 2004, Lammi *et al*.[16] showed that a nonsense mutation in *AXIN2*(c.1966C>T, p.Arg656Stop) caused oligodontia and predisposed to cancer. Additionally, in 2009, Marvin *et al*.[37] described a family with a novel, nonsense *AXIN2* mutation (c.1989G>A, p.Tyr663X) segregating in an autosomal dominant pattern with oligodontia and variable other findings, including colonic polyposis, gastric polyps, a mild ectodermal dysplasia phenotype with sparse hair and eyebrows, and early onset colorectal and breast cancers. In the remaining five *AXIN2* mutations causing non-syndromic tooth agenesis, four are missense mutations (p.Ala758Thr, p.Ala684Val, p.Thr308Met and p.Met830Ile), and one is a frameshift mutation (p.Gly666GlyfsX42) [6,16,21]. It appears that *AXIN2* mutations that lead to a truncated AXIN2 protein are more likely to lead to syndromic oligodontia and predispose to cancer. In this study the missense mutation in AXIN2 (c.314T>G) resulted in the substitution of Val at residue 105 for Gly. Structural analysis predicted that the 3D structures of the wild-type and mutant RGS domain of AXIN2 are different at residue 105 (Fig 4). This results suggests that although the overall structure of the protein is complete, the p.Val105Gly substitution may affect some functional activity of AXIN2 protein. This may lead to the occurrence of clinical symptoms. Of course, functional study of the mutant protein need to be done to confirm the assumption in the future. Because the *AXIN2* mutation (c.314T>G) identified in the present study occurred *de novo*, cancer prevention measures are necessary for the 8-year-old male proband.

Interestingly, in the present study, the proband was missing a total of seven permanent teeth (excluding third molars), all of which were premolars. In previous studies, the positions of missing teeth in patients with *AXIN2* mutation were not identical [6,16,21,37]. At present, the phenotype-genotype association in AXIN2-related patients is unclear. However, in all cases, patients with an *AXIN2* mutation were missing at least seven teeth. This indicates that any change in the AXIN2 protein can cause severe tooth agenesis.

In conclusion, this study report describes a *de novo AXIN2* missense mutation (c.314T>G) in a Chinese individual with non-syndromic oligodontia. This mutation is located at the regulator of G protein signaling (RGS) domain of the AXIN2 protein, which is a highly conserved region. In addition, this study extends the mutation spectrum of the *AXIN2* gene in individuals with non-syndromic tooth agenesis. Furthermore, our findings confirm that *AXIN2* plays an important role in human tooth development. However, further studies are necessary to clarify the precise role of *AXIN2* in tooth development.
